# Frost Damage Mitigation in Flowers and Fruitlets of Peach and Almond from the Application of a Multi-Attribute Approach Biostimulant

**DOI:** 10.3390/plants13121603

**Published:** 2024-06-08

**Authors:** Estanis Torres, Xavier Miarnau

**Affiliations:** Fruit Production Program, IRTA-Institute of Agrifood Research and Technology, Park Agrobiotech, Fruitcentre Building, E-25003 Lleida, Spain; xavier.miarnau@irta.cat

**Keywords:** plant frost protection product, α-tocopherol, glycol, boron, freeze injury, lethal temperature, *Prunus dulcis*, *Prunus persica*

## Abstract

To prevent frost damage in fruit trees, growers employ passive and active methods, and one of these second methods is the use of biostimulant compounds against abiotic stress. In this study, two trials were conducted to evaluate the effectiveness of a multi-attribute approach biostimulant—containing α-tocopherol, boron, and glycols, in peach (‘UFO-4’ cultivar) and almond (‘Vairo’ cultivar) trees. In a first trial, one-year-old shoots with flowers were collected after 24 h, 48 h, and 96 h of the biostimulant applications. Two different application rates of the product (1000 and 2000 cc ha^−1^) were tested and compared to an untreated control. In a second trial, one-year-old shoots with fruitlets were collected after 24 h of the biostimulant applications. In this case, only an application rate (2000 cc ha^−1^) was tested. In the two trials, the collected one-year-old shoots were subjected to different frost temperatures using a controlled environment chamber. The damage level was assessed by a morphological analysis of the flowers and fruitlets 96 h after each frost cycle simulation. The lethal temperatures (LT_10_, LT_50_, and LT_90_) of each treatment were calculated by probit analysis. The product applied 24 h and 48 h before the frost simulations significantly decreased the LT_10_ and LT_50_ in 1.5 °C in peach flowers, and 2.5 °C in almond flowers (a temperature reduction of 50% and 75%, respectively). These results were more consistent when the application volume was 2000 cc ha^−1^, instead of 1000 cc ha^−1^. Significant differences between treated and non-treated fruitlets were observed only in almond fruitlets, with LT_10_ and LT_50_ being 0.5 °C lower in treated fruitlets (20% reduction). In conclusion, the multi-attribute approach biostimulant applied 24 or 48 h before the frost reduced the mortality of peach and almond flowers, but its effectiveness to protect fruitlets after bloom was inconsistent.

## 1. Introduction

An important cause for lack of fruit production in extreme weather areas is frost damage. Frost or freeze events around bloom and fruit set can significantly reduce fruit production and crop value in fruit orchards and trigger huge economic losses. As examples, a single frost night on 19 April 2017 led to apple yield losses of up to 78% in Europe compared to the preceding seven years [1]. In Spain, spring frosts in 2022 caused almond and peach yield losses of around 50–80%, resulting in serious economic and social problems in the main fruit production areas [2]. Most studies of the climate models and assessment methodologies confirm that frost during flowering will remain a major risk factor for fruit production in warming temperate regions [3].

Frost-induced damage causes biophysical changes. Tissues are damaged by frost due to ice formation inside the cells. Ice crystals inside the cytoplasm destroy the intracellular structures and the cells lose their functionality [4]. This damage results in necrotic tissues, which can be readily identified from their brown color. Young and soft plant tissues are more susceptible than mature tissues. Hence, reproductive tissues, such as flowers and fruitlets, are more sensitive to freeze damage than the vegetative organs [5]. Additionally, when determining the susceptibility to frost damage, bloom time, and fruit set are important parameters since early blooming cultivars are more exposed to frost conditions. Thus, due to early blooming, peaches (*Prunus persica* (L.) Batsch) and almond (*Prunus dulcis* (Mill.) D.A. Webb. syn. *P. amygdalus* (L.) Batsch) trees can be more easily harmed by early frost, or even by late seasonal frost, than other late blooming fruit trees such as apple, pear, or cherry trees. Peach is the main flesh fruit grown in Spain. They are grown mainly in the Ebro Valley and Murcia, but also throughout the Mediterranean area and Extremadura. Peach varieties have been bred for southern climates to bloom sooner, during early seasonal time windows, in order to harvest earlier and take advantage of high prices at the beginning of the season. In the last years, there has been a deep shift in the Spanish almond sector, with new varieties and an increase in the number of irrigated plantations with highly professionalized management. All this has resulted in a significant increase in the production of almond in Spain. Late flowering has been a key trait in new almond cultivars to avoid damage in cold areas [6]. However, due to the expansion of almond cultivation, late-flowering cultivars are also being grown in newly irrigated areas of Mediterranean countries with a high-frost risk.

Several methods for frost protection are available, such as over-head sprinklers, heaters, fogging systems, or wind machines. However, the main disadvantage of these approaches is the availability of water, high-energy costs, and environmental concerns. The application of chemical products for crop protection against freezing stress has been also suggested as approach to reduce frost damage, which can be used together with the other methods cited above [7]. Among this type of chemical products, compounds with antioxidative properties have demonstrated to be effective at reducing different types of abiotic stress-induced damages in plants [5,8,9,10]. In general, most abiotic stress factors such as heat, solar radiation or frost induce the formation of reactive oxygen species (ROS) and free radicals in the plant tissue [11]. ROS such as H_2_O_2_ or O^2−^ cause the oxidation of cell membranes in a chain reaction and finally lead to cell death [12]. ROS has harmful effects such as DNA damage, oxidation of amino acids (AAs) and proteins, and lipid peroxidation. In the latter process, lipids are broken, which affects their function on the membrane, causing loss of fluidity, and inactivation of membrane enzymes [9]. Plants respond to oxidative stress with the formation of antioxidants such as α-tocopherol (vitamin E) or ascorbic acid (vitamin C), carotenoids and phenolic compounds that are able to eliminate ROS and free radicals and prevent the cell membranes from disintegration [13]. Consequently, the exogenous application of compounds with anti-oxidative properties can provide protection against frost damage [14,15]. Among these anti-oxidative compounds, the use of α-tocopherol has been shown to be effective in protecting crops against freezing since, additionally to anti-oxidative properties, it can protect the integrity of the cell membrane [7]. The α-tocopherol is the main component of vitamin E, which is a lipophilic antioxidant that acts against free radicals induced by freezing temperatures [10]. Furthermore, α-tocopherol is incorporated in membranes, contributing to their biophysical stability and functioning [8].

Other kinds of compounds used to protect plants against frost are cryoprotectants. They are used to prevent ice formation, which causes freezing damage to the biological tissue when cooling the organs. Cryoprotectants such as glycol, glycerol, and dimethyl sulfoxide (DMSO) are able to lower the freezing point when applied via spraying on different crops [10,14,16,17]. They reduce the ice formation by increasing the total concentration of all the solutes on dissolving in water. When a slow freezing rate occurs, there is sufficient time for intracellular water to move out of cells under the osmotic pressure, resulting in a reduction in cell volume. To deal with this problem, permeable cryoprotectants that can enter cells can be used to induce the vitrification of intracellular environment before ice crystal formation, thus preventing excessive loss of cell volume [18]. Mineral nutrients, such as nitrogen, potassium, and calcium, can also to protect biological tissue against low-temperature stress by increasing the total concentration of all the solutes present in the system and improve the structures and properties of cell membrane. Among evaluated mineral nutrients, boron has been proven effective in enhancing the frost tolerance of plants by stabilizing the cell wall [19]. As example, Räisänen, et al. [20] improved frost tolerance after treatments with boron-containing sprays in shoots of Norway spruce (*Picea abies* L.) trees. Furthermore, Rufat and Arbonés observed that foliar boron sprays in almonds delayed bloom by around 6 days [21], which may reduce the possibility of blossom damage from late winter or early spring frosts.

From the above-mentioned scenarios, the use of biostimulant compounds containing α-tocopherol, glycols, and mineral nutrients such as boron could be an alternative to protect crops from frost. While α-tocopherol eliminates free radicals and cryoprotectants prevent the extracellular formation of ice, boron plays a major role in regulating the permeability of cell membranes [22,23]. Likewise, the AAs usually applied in biostimulants are used by plants to increase the biosynthesis of several nitrogenous compounds such as coenzymes, vitamins, pigments, and pyrimidine bases [24,25]. Several authors suggest the participation of AAs in reducing abiotic stress as precursors of hormones and N carriers [26]. Although the use of the above-mentioned compounds to protect frost damage has been reported for different crops such apples [14], tomatoes [17], or potato [27], more studies in field-crop research to know their effectiveness are necessaries, especially in the case of products that combine different mechanisms which need to be explored in more detail [7].

The aim of the present study was to study the potential of a multi-attribute approach biostimulant compound, based on the above-mentioned components (α-tocopherol, glycol, and boron), to prevent frost damage to flowers and fruitlets of peach and almond under frost-controlled conditions. This study aims to assess the level of effectiveness according to the freezing temperature, phenological stage, dosages, and days between the application and the frost event, calculating lethal temperatures (LTs) and their fiducial confidence limits for every case from probit analysis, as well as both the equality and the parallelism hypotheses for the probit lines.

## 2. Material and Methods

### 2.1. Plant Material and Trial Location

The peach orchard was in the municipality of Gimenells (41°39′18.77″ N and 0°23′31.41″ E), in the province of Lleida (Catalonia, Spain). The assays were performed on ‘UFO-4’ peach cultivar, grafted onto ‘Garnem’ an almond x peach hybrid rootstock, planted in June 2007, at 5 × 3 m and formed as a classical vase. UFO-4 is a flat-shape, white-flesh peach variety with low chilling requirements that is harvested early in the season; the tree has semi-open behavior, strong growth, its flowering is rich and occurs in the middle of the season. The almond orchard was planted in June 2009, in the municipality of Les Borges Blanques (41°30′31.89″ N; 0°51′10.70″ E) in the province of Lleida (Catalonia, Spain). The assays were carried out on ‘Vairo’, a high vigor, late-flowering and self-pollinating almond cultivar, grafted onto ‘INRA GF-677’ an almond x peach hybrid rootstock, planted at 5 × 3 m and formed as a central axis. The trees of both orchards were in full production (more than 5 years old), with a good balance of vegetation–production.

The soil of the peach and almond orchards was calcareous, alkaline, non-contaminated and non-saline with clay loam texture, good water-holding capacity, well drained, and fertile with about 2% organic matter content.

The climate of the fields sites area is semi-arid Mediterranean, Bsk according to the Köppen–Geiger climate classification system, with a mean annual rainfall of 350 mm and a mean summer daily temperature of 32 °C.

Both orchards were managed with drip irrigation system, and the cultural practices, such as pruning, soil management, nutrient supply and phytosanitary protection were performed under the guidelines of Spanish integrated pest management practices.

### 2.2. Treatments and Experimental Design

Two experiments in each crop were conducted to evaluate the effect of the treatments on the frost damages at different phenological stages (Table 1): on flowers (experiment 1) and fruitlets (experiment 2).

Experiment 1: In March 2014, two application dosages (1000 and 2000 cc ha^−1^ of compound) were evaluated in each crop when 50–75% of the flowers were fully open (phenological growth stage BBCH 65). For both dosages, we prepared a solution 200 cc hL^−1^ which was applied at a volume of 500 and 1000 L ha^−1^, respectively. The applications were made as foliar sprays applied early in the morning using a high-pressure handgun sprayer (25 atm, Gaysa, Librilla, Spain). An untreated control (UTC) was included using tap water instead of product solutions. To assess the product persistence after application, each treatment was subjected to five frost cycles (−1, −3, −5, −7, and −9 °C, see frost cycles simulations) after 24, 48, and 96 h after application. Indeed, the objective of the experiment 1 was to determine if there was an interaction effect between the three independent variables: cultivar (peach and almond), dosage (1000 and 2000 cc ha^−1^), and time (24, 48, and 96 h after application) on the frost damage.

Experiment 2: According to results of experiment 1 in April 2015, one application at 2000 cc ha^−1^ was assessed in peach and almond trees when all fruitlets had 100% jacket split (phenological growth stage BBCH 72; fruit diameter ~10 mm). UTC was included using tap water. Each treatment was subjected to six frost cycles (−1, −3, −5, −7, −9, −11 °C, see frost cycles simulations) only after 24 h.

### 2.3. Frost Cycles Simulations

Five (experiment 1) and six (experiment 2) frost cycles were simulated at different frost objective temperatures to obtain a gradient of frost damage which allows a proper analysis of the data [28]. For each frost cycle simulation and treatment (including UTC), three replications of five one-year-old shoots (OYOS) of similar length and the same height of the tree were taken from three treated trees. Once cut, they were transported to IRTA facilities at Lleida where were stored at 6 °C until they were subjected to the corresponding frost cycle. Finally, a total of around 750 flowers or fruitlets were evaluated in each frost cycle.

Frost cycles in experiment 1 were simulated using a controlled environment chamber IBERCEX, V-1300-B model. The chamber contained three trays at different heights. Each of these trays was used as replication of each treatment corresponding to one 5-OYOS replicate (total 15 OYOS). Frost cycles in experiment 2 were simulated using a controlled environment chamber of ARALAB, FITOCLIMA 10000HP model. Three 5-OYOS replicates were placed inside the chamber at 1.5 m above ground level, on perforated movable tables allowing the circulation of airflow inside the camera.

**Figure 1:**
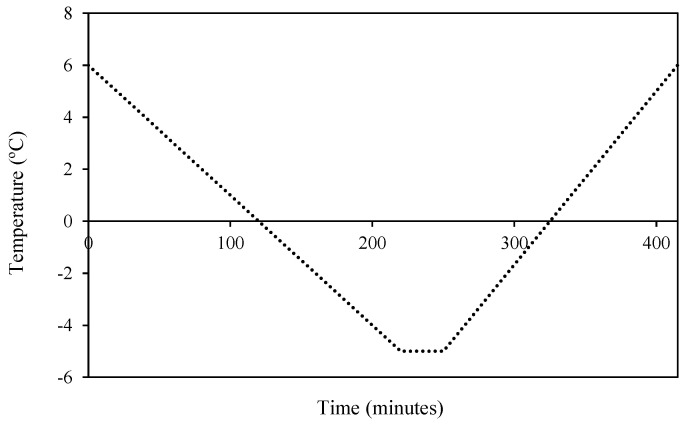
Example of a frost cycle simulated in the climatic chamber (30 min at −5 °C).

Each frost cycle simulation consisted in different steps according to the method used by Miranda et al. [28]. First, the air temperature in the chamber was acclimated to 6 °C for 30 min, after which the air temperature dropped gradually by 2–3 °C per hour until it reached the desired frost temperature (FT). Then, the FT remained constant for 30 min. Finally, the temperature began to increase by 3–4 °C per hour, until it reached 6 °C (Figure 1). Finally, the samples were removed and stored in a cold room at 6 °C until the evaluation of damages after 96 h.

### 2.4. Frost Damage Evaluation

**Figure 2:**
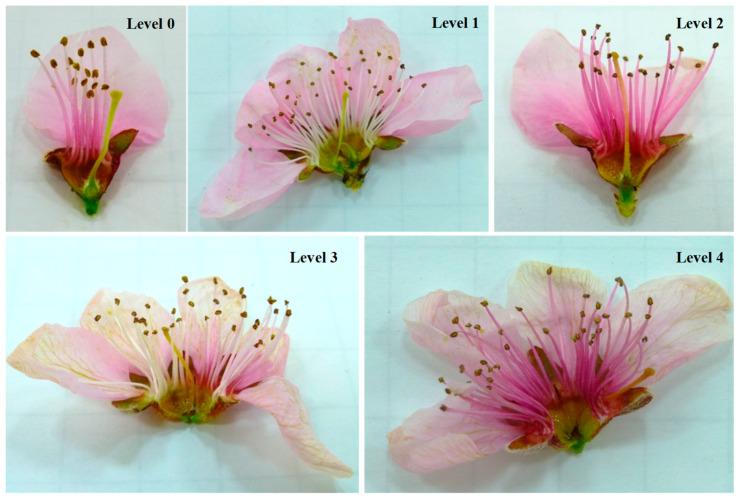
Levels of frost damage in peach flowers (UFO-4), according to the scale from level 0 (**top-left**) to level 4 (**bottom-right**) used in the damage severity assessment.

**Figure 3:**
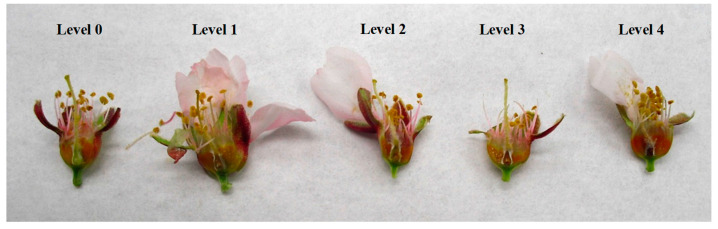
Levels of frost damage in almond flowers (Vairo), according to the scale from level 0 (**left**) to level 4 (**right**) used in the damage severity assessment.

**Figure 4:**
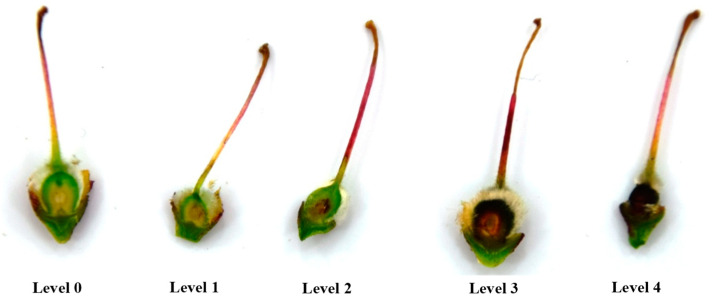
Levels of frost damage in peach fruitlets (UFO-4), according to the scale from level 0 (**left**) to level 4 (**right**) used in the damage severity assessment.

**Figure 5:**
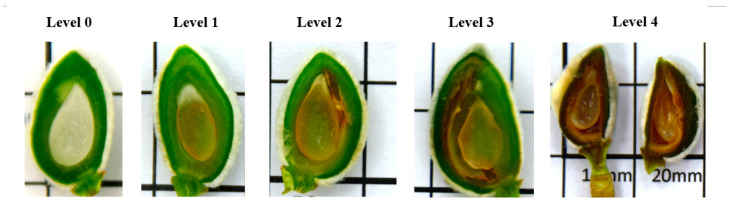
Levels of frost damage in almond fruitlets (Vairo), according to the scale from level 0 (**left**) to level 4 (**right**) used in the damage severity assessment.

Flower and fruitlets were cut, open and inspected to determine if they were frost damaged after a frost cycle. Flowers and fruitlets having necrosed pistils and/or ovaries indicated they had been damaged, and fruit will not form. For flower damage assessment, five levels were established (Figure 2 and Figure 3): level 0 (no damage), level 1 (basal part of the pistil damaged), level 2 (internal side of ovary damaged), level 3 (seminal primordia damaged), and level 4 (additional external ovary area damaged). The flowers were dissected and observed in a Leica MZ 16 F stereomicroscope (Leica Microsystems Ltd., Heerbrugg, Switzerland). For fruit damage assessment, five categories were also established (Figure 4 and Figure 5): level 0 (no damage), level 1 (nucela and integument damaged), level 2 (endocarp and kernel damaged), level 3 (endocarp and mesocarp damaged), and level 4 (whole fruit damaged). The incidence of damaged flowers or fruitlets in one-year-old shoots was calculated as the percentage of flowers or fruitlets with an affectation level ≥ 1. Additionally, the severity relative level (SRL) was also calculated for each OYOS from the following formula:

$$SRL=\frac{\sum_{n=1}^{L}I_{n}}{4L}$$

where *S* is the relative level of severity, *I* is the index of severity of damage each flower or fruitlet, ‘4’ is the highest level of severity of the used scale and *L* is the number of flowers or fruitlets evaluated.

### 2.5. Statistical Analysis

Statistical analyses for estimation of lethal temperatures (LT) causing 10% (LT_10_), 50% (LT_50_) and 90% (LT_90_) mortality were performed using probit analysis on the PoloPlus software version 1.0 (LeOra, 2004). This procedure computed approximate standard errors for the estimates using the delta method and these standard errors were used to compute corresponding 95% confidence limits. Non-linear modeling was used to estimate temperature–mortality relationships by fitting the binomially distributed mortality data to the mortality rate (*m*) formula (probit regression model):

*m* = *c* + (1 − *c*) *Φ* (*a* + *bt*),

where *c* is the control mortality rate (flowers or fruitlets level ≥ 1), *Φ* (phi) is the cumulative normal probability function, *b* determines the slope and *t* is the temperature (negative Celsius degrees, −°C). Inferences about treatment effects were made by the hypothesis of equality and parallelism between lines of different treatments using chi-square test. Corresponding 95% confidence limits were compared to evaluate equality hypothesis between treatments. If the limits overlapped, then it was considered that the critical temperatures did not differ significantly except under unusual circumstances [29].

Differences in tolerance to freeze-induced damage were also tested by comparing the *S* using a three-way ANOVA (dosage, time, and temperature) in the experiment 1 and a one-way ANOVA in the experiment 2, for each crop separately. In these tests, *S* values were arcsine square root transformed to improve normality and homogeneity of variance. A post hoc Duncan’s multiple-range test was used to separate significantly different groups using SAS program package (SAS Institute, Cary, NC, USA, 2008).

## 3. Results

### 3.1. Experiment 1

#### 3.1.1. Peach Blossom

**Figure 6:**
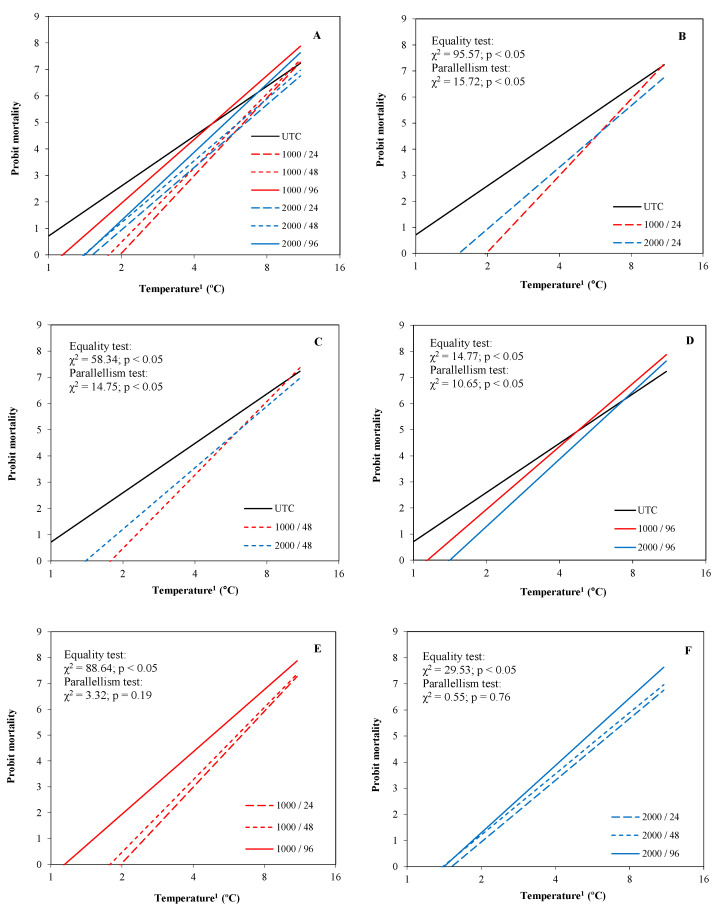
Probit regression lines between mortality and temperature of each treatment applied on peach flowers (experiment 1). (**A**): All treatments; (**B**): UTC, and the treatments at 1000 and 2000 cc ha^−1^ applied 24 h before the frost simulation; (**C**): UTC, and the treatments at 1000 and 2000 cc ha^−1^ applied 48 h before the frost simulation; (**D**): UTC, and the treatments at 1000 and 2000 cc ha^−1^ applied 96 h before the frost simulation; (**E**): treatments at 1000 cc ha^−1^ applied 24, 48, and 96 h before the frost simulation; (**F**): treatments at 2000 cc ha^−1^ applied 24, 48, and 96 h before the frost simulation. ^1^ Absolute values of temperature in logarithmic scale (base 2).

According to probit model, the effect of the treatments was higher in the first ranges of temperature below 0 °C, whereas no effect was observed at extreme low temperatures (beyond −8 °C, Figure 6A). The probit lines of the treatments applied at 24 (Figure 6B), 48 (Figure 6C) and 96 (Figure 6D) hours before frost (HBF) were significantly different to those for UTC. The treatments applied at 24 and 48 HBF showed the greatest differences compared to UTC. In addition to this, both application times showed probit lines significantly different compared to the treatments applied at 96 HBF and without significant differences between them (Figure 6E,F). Regarding the dosages, no significant differences were observed between the applications at 1000 and 2000 cc ha^−1^, independently of the time of application. However, it is worth highlighting that the slope and the ordinate at the origin of the treatment applied at 96 HBF tended to decrease when it was applied at 2000 cc ha^−1^, indicating a greater persistence of the efficacy compared to 1000 cc ha^−1^ (Figure 6D).

The treatments applied at 24 and 48 HBF significantly reduced the LT_10_ and LT_50_ compared to UTC, independently of the dosage. The LT_10_ was reduced between 29 and 36% and the LT_50_ between 21 and 27%. There were no significant differences between applying at 24 and 48 HBF for the LT_10_ and LT_50_ (Table 2). For the LT_90_, the values were significantly reduced compared to UTC only when the treatments were applied at 48 or 24 HBF at 2000 cc ha^−1^ (about 13–19% less). The treatments at 96 HBF obtained significant differences compared to UTC only when they were applied at 2000 cc ha^−1^ for the LT_10_ (Table 2).

**Figure 7:**
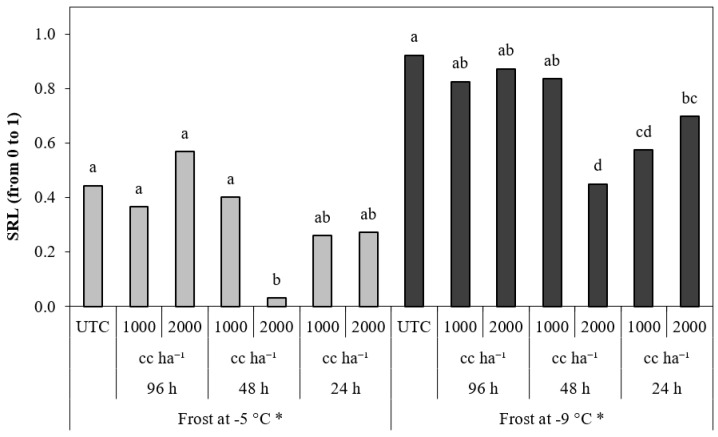
Severity relative level (SRL) in peach flowers for the frost cycle simulations at −5 °C, −7 °C and −9 °C of frost objective temperature. * Within the same frost cycle, treatments with different letters are significantly different according to the Duncan test (*p* < 0.05).

Regarding the SRL, the differences between treatments were significant for the simulation at −5 and −9 °C (Figure 7). The treatment applied at 24 HBF, independently of the dosage, obtained a SRL less than that obtained by UTC when the temperature fell to −9 °C, and the treatment applied at 48 HBF at 2000 cc ha^−1^ obtained a SRL less than that obtained by UTC when the temperature fell to −5 and −9 °C.

#### 3.1.2. Almond Blossom

**Figure 8:**
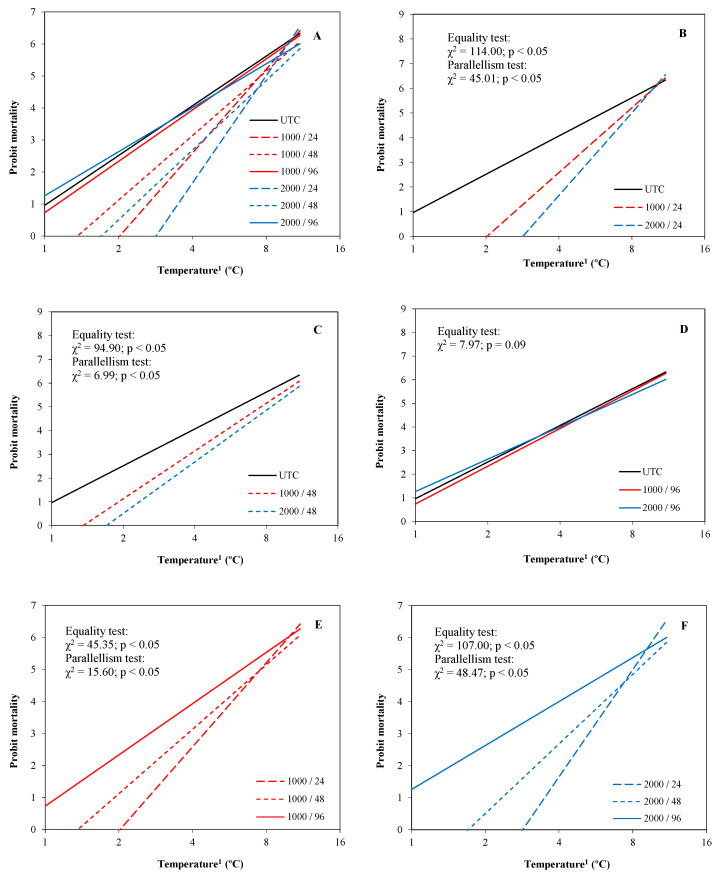
Probit regression lines between mortality and temperature of each treatment applied on almond flowers (experiment 1). (**A**): All treatments; (**B**): UTC, and the treatments at 1000 and 2000 cc ha^−1^ applied 24 h before the frost simulation; (**C**): UTC, and the treatments at 1000 and 2000 cc ha^−1^ applied 48 h before the frost simulation; (**D**): UTC, and the treatments at 1000 and 2000 cc ha^−1^ applied 96 h before the frost simulation; (**E**): treatments at 1000 cc ha^−1^ applied 24, 48, and 96 h before the frost simulation; (**F**): treatments at 2000 cc ha^−1^ applied 24, 48, and 96 h before the frost simulation. ^1^ Absolute values of temperature in logarithmic scale (base 2).

Like peach trial, treatments applied at 24 and 48 HBF achieved a tolerance to frost significantly higher than UTC, with probit models significantly differing according to the hypotheses of equality and parallelism (Figure 8B,C). Their slope and abscissa of origin in absolute terms were greater than those of UTC, indicating a greater effectiveness in the first temperature ranges below zero. However, the differences reduced quickly when the temperature fell below −8 °C (Figure 8B,C). No significant differences were observed between UTC and the treatments applied at 96 HBF (Figure 8D). In contrast to peach results, significant differences between the applications at 24 and 48 HBF were observed, as well as between applications at 1000 and 2000 cc ha^−1^ (Figure 8E,F). The probit lines of the treatments at 24 HBF registered a higher ordinate at the origin in absolute terms than those from the treatments at 48 HBF, indicating a greater effect at 24 HBF than at 48 HBF. Similarly, the treatments at 2000 cc ha^−1^ showed a higher ordinate at the origin in absolute terms than those from the treatments at 1000 cc ha^−1^, indicating a greater effect at 2000 cc ha^−1^ than at 1000 L/ha cc ha^−1^.

The treatments applied at 24 and 48 HBF significantly decreased the LT_10_ and LT_50_ compared to UTC, independently of the dosage (Table 3). In general, the treatments applied at 24 and 48 HBF presented a reduction in LT_10_ and LT_50_ of 37–44% and 20–27%, respectively. Additionally, the treatments applied at 24 HBF tended to obtain better results than those obtained when applying 48 HBF, but without significant differences between a same application dose. A significant response to dosage was observed in the reduction in LT_10_ and LT_50_ when applying 24 or 48 HBF. Indeed, increasing the application dose from 1000 to 2000 cc ha^−1^ reduced LT_10_ by 16% and LT_50_ by 10%. Compared with UTC, the treatments at 1000 cc ha^−1^ presented a reduction in LT_10_ and LT_50_ of 36% and 22%, respectively, while at 2000 cc ha^−1^ the reductions were of 42% and 26%, respectively. No significant differences between treatments were observed for LT_90_, independently of the application time and dosage.

**Figure 9:**
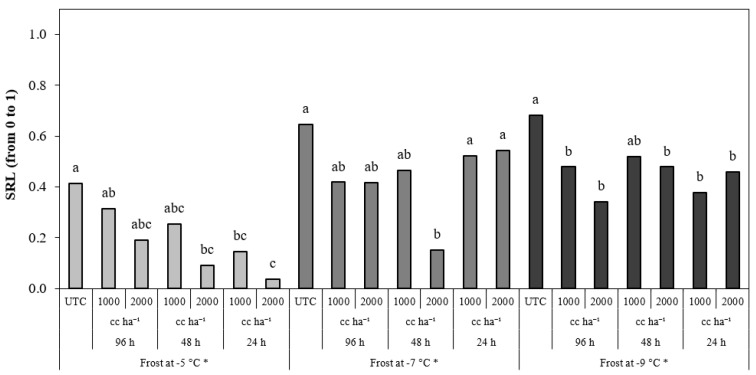
Severity relative level (SRL) in almond flowers for the frost cycle simulations at −5 °C, −7 °C, and −9 °C of frost objective temperature. * Within the same frost cycle, treatments with different letters are significantly different according to the Duncan test (*p* < 0.05).

Significant differences between treatments were observed for the SRL when the temperatures fell to −5, −7, and −9 °C (Figure 9). The treatments applied at 24 HBF obtained a SRL significantly less than UTC in the cycles at −5 and −9 °C, independently of the application dose. The treatments applied 48 HBF obtained a SRL significantly less than UTC in the cycles at −5, −7, and −9 °C, but the differences were statistically significant only when they were applied at 2000 cc ha^−1^. The treatments applied at 96 HBF obtained a SRL significantly less than UTC when the temperature fell to −9 °C, independently of dosage.

### 3.2. Experiment 2

#### 3.2.1. Peach Fruitlets

**Figure 10:**
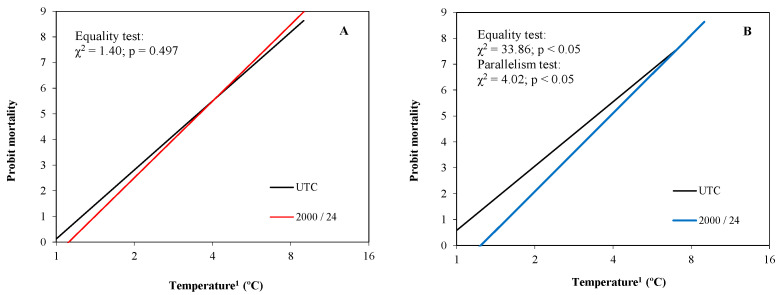
Probit regression lines between mortality and temperature of each treatment of experiment 2 in peach fruitlets (**A**) and almond fruitlets (**B**). ^1^ Absolute values of temperature in logarithmic scale (base 2).

No significant differences between treatments were observed in the assessments carried out on peach fruitlets (Figure 10A). According to the chi-square test, no significant differences were observed between the probit models, either for the hypothesis of equality or parallelism. No significant differences between treatments were observed for the LT_10_, LT_50_, and LT_90_ and the differences with respect to UTC were less than −0.2 °C in any case (Table 4). No significant differences between treatments were observed for the severity values.

#### 3.2.2. Almond Fruitlets

Contrary to peach, significant differences between treatments were observed in almond fruitlets (Figure 10B). The hypothesis of equality and parallelism between the probit lines were rejected according to the chi-square test, with an ordinate at the origin in absolute terms of the probit line from the treated fruitlets greater than those of UTC, indicating mortalities lower than UTC to −4.0 °C. No significant effect of treatment was observed when temperatures fell below −6.0 °C. Finally, treated fruitlets obtained a LT_10_ and LT_50_ around 0.5 °C inferior to those of UTC, with significant differences between treatments (Table 4). No significant differences were observed for the LT_90_, although the mean value for the treated samples was 0.3 °C lower than those of UTC. No significant differences between treatments were observed for the severity values.

## 4. Discussion

Herein, the probit model was used to evaluate the effectiveness of a plant frost protection biostimulant against freezing stress in peach and almond crops. In the field of agricultural sciences, the probit model has been previously applied, especially in the fields of plant pathology [30,31] and entomology [32]. An additional benefit of the probit model for the study of plant frost protection products is that its effectiveness can be evaluated directly determining the LTs for the probability value desired. Proebsting and Mills were pioneers in temperature stress research for estimating lethal temperatures in deciduous fruit species [33]. Since then, other authors have contributed to knowledge of frost or lethal temperatures in different species from logit or probit models [28,34,35,36]. However, to our knowledge, there are no earlier reports on using the probit model to study the effectiveness of PFPPs. Hereby, the LT_10_, LT_50_, and LT_90_, with associated confidence intervals, were easily estimated from different probit models for different treatments. Our results suggest that the treated flowers were more frost resistant than untreated flowers. In general, the application of the tested treatments increased the LT_10_ of peach flowers from −3.0 °C to −4.5 °C and the LT_50_ from −4.8 °C to −6.3 °C when they are applied at 24–48 HBF. In the case of almond flowers, the differences were still greater, and increased the LT_10_ from −3.4 °C to −5.5 °C and the LT_50_ from −6.1 °C to −7.9 °C.

According to the literature, the use of plant growth regulators, anti-transpirants, or mineral nutrients as plant frost protection products have shown inconsistent and sometimes contradictory results [7,37,38,39,40]. On the other hand, alcohols (glycol and glycerol), α-tocopherol and other lipids (especially phospholipids) have showed positive results when they have been evaluated separately or in combination in different crops such as apple flowers [14,41,42], peach flowers and fruitlets [42], common bean, and potato [27]. The application of cryoprotectants as Basfoliar^®^ Frost Protect, which contains α-tocopherol and glycols, has been shown to effectively mitigate ROS toxicity in plant cells [15]. α-Tocopherol and phospholipids improve the stability and integrity of the cell membrane, which improves freezing tolerance [43,44]. Glycols (polyols) and glycerol act as solutes or osmolytes that diminish the freezing point, both inside and outside plant cells [45,46,47]. The results against freezing damage in flowers and fruitlets could be improved by combining these components [7]. In addition to these components, the multi-attribute approach biostimulant studied herein is based on the mixture with boron. Historical reports of possible boron roles in the protection of tree and horticulture species against frost damage have been reported from the early 1950s [23]. Boron regulates the orientation of pectin chains in cell walls by forming bonds between rhamnogalacturonan-II chains affecting the membrane rigidity [20,22,23,48]. Furthermore, boron supplementation can improve low-temperature tolerance because a high solute quantity reduces the water freezing temperature.

Our results from experiment 1 showed a significant decrease in LT_10_, LT_50_, and, in some cases, LT_90_, as well as minor damage to peach and almond flowers when the sprayings were performed at 24 or 48 HBF. On the other hand, no significant effect was observed when the spraying was performed at 96 HBF. To the best of our knowledge, this was the first experimental evaluation where α-tocopherol and glycol or glycerol against freezing stress are evaluated beyond 48 h after application, confirming a lack of efficacy this time. In general, no significant differences between applying 24 and 48 HBF were observed, although the applications at 24 HBF in almond flowers tended to show better results. These results agree with those obtained by Hołubowicz, Basak, and Pacholak who increased the survival of flowers in ‘Reliance’ peach trees spraying with a mixture based on vitamin E and C with glycerol, and without significant differences between spraying at 24 and 42 HBF [42].

In general, the highest dosage at 2000 cc ha^−1^ tended to show better results compared to the dosage at 1000 cc ha^−1^, especially in almond flowers where increasing the dosage meant a significant reduction in LT_10_ and LT_50_ and a greater persistence as indicated the differences between 24 and 48 HBF observed for the SRL. Therefore, according to these results, the applications at 2000 cc ha^−1^ 24 HBF ensure a significant efficacy in both crops at bloom stage if frost temperatures do not fall below −7 °C. Hence, an application dose and time of 2000 cc ha^−1^ and 24 HBF, respectively, were chosen for experiment 2 in fruitlets. In general, the efficacy after bloom in fruitlets decreased compared to the results at bloom stage. Indeed, a slight but significant effect was obtained only in almonds fruitlets, but no significant effect was observed in peach fruitlets. Hołubowicz, Basak, and Pacholak also observed a decrease in effectiveness in peaches and apples when spraying at more advanced phenological stages [42].

The differences observed in this study between both phenological stages (flowers and fruitlets), and crops (peach and almond), could be due to a decrease in freezing tolerance at more advanced stages and genetic differences in freeze tolerance, respectively [28]. The timing and intensity of changes in fruit water content and solute content are highly genotype dependent [49]. During fruit growth, increased water content occurs through cellular vacuolization and additional water is accumulated in the pectin fraction of cell walls [49]. This directly results in an increase in fruit volume and in the loss of the tolerance to freezing temperatures [50]. Differences between both species in the flower anatomy can also have a relevant role. Montañés Millán, et al. found significant differences between flowers of peaches and almonds for the fresh and dry weight which could be related with the water content and, consequently, tolerance to freezing stress [51]. In conclusion, the blending of α-tocopherol with glycol and boron can reduce the mortality of peach and almond flowers but inconsistent results were obtained in fruitlets after bloom.

## Figures and Tables

**Table 1:** Experiments, treatments, doses, application volumes, dosage, application times, phenological stages, and tested frost objective temperatures per frost cycle simulations.

Experiment	Treatment	Dose ^(1)^ (cc hl^−1^)	Application Volume (L ha^−1^)	Dosage (cc ha^−1^)	Application Time ^(2)^ (HBF)	Phenological Stage (BBCH Scale)	Frost Objective Temperature (°C)
1	UTC ^(3)^	-	1000	-	-	50–75% full bloom (BBCH 65)	−1; −3; −5; −7; −9.
	1000/24	200	500	1000	24		
	2000/24		1000	2000			
	1000/48		500	1000	48		
	2000/48		1000	2000			
	1000/96		500	1000	96		
	2000/96		1000	2000			
2	UTC ^(3)^	-	1000	-	24	100% jacket split ^(4)^ (BBCH 72)	−1; −3; −5; −7; −9; −11
	2000/24	200		2000			

^(1)^ Commercial product: Basfoliar Frost Protect^®^ (α-tocopherol, glycol, boron). ^(2)^ HBF: hours before frost. ^(3)^ UTC: untreated control (tap water without commercial product). ^(4)^ Treatments were made at 10 mm fruit diameter.

**Table 2:** Probit analysis results in peach (Experiment 1): treatments, number of evaluated flowers, slopes, lethal temperatures when the mortality rate reached 10%, 50%, and 90% of flowers (LT_10_, LT_50_, and LT_90_, respectively) and ratio between the LT_50_ of treated flowers, and the LT_50_ of untreated control (UTC).

Treatment	Num. Flowers	Slope (±SE)	LT_10_ (FL at 95%) *	LT_50_ (FL at 95%) *	LT_90_ (FL at 95%) *	Ratio LT_50_
UTC	761	6.26 ± 0.39	−3.0 (−2.6/−3.3) a	−4.8 (−4.6/−5.1) a	−7.8 (−7.2/−8.3) ab	-
1000/24	702	9.75 ± 0.91	−4.7 (−4.3/−5.1) b	−6.4 (−6.1/−6.7) b	−8.7 (−8.3/−9.3) bc	0.79
2000/24	735	7.86 ± 0.69	−4.5 (−4.1/−4.8) b	−6.6 (−6.2/−6.9) b	−9.6 (−8.9/−10.6) c	0.79
1000/48	823	9.31 ± 0.81	−4.5 (−4.1/−4.8) b	−6.1 (−5.9/−6.4) b	−8.4 (−8.0/−9.0) bc	0.79
2000/48	572	7.78 ± 0.77	−4.2 (−3.7/−4.6) b	−6.1 (−5.7/−6.5) b	−9.0 (−8.3/−10.0) c	0.79
1000/96	768	8.01 ± 0.58	−3.3 (−3.1/−3.5) a	−4.8 (−4.6/−5.0) a	−7.0 (−6.5/−7.6) a	1.01
2000/96	623	8.56 ± 0.80	−3.8 (−3.4/−4.2) b	−5.4 (−5.0/−5.7) a	−7.6 (−7.2/−8.2) ab	0.89

* LTs with the same letter are not significantly different by fiducial limits (FL) overlapping (*p* = 95%).

**Table 3:** Probit analysis results in almond (Experiment 1): treatments, number of evaluated flowers, slopes, lethal temperatures when the mortality rate reached 10%, 50% and 90% of flowers (LT_10_, LT_50_, and LT_90_, respectively), and ratio between the LT_50_ of treated flowers and the LT_50_ of untreated control (UTC).

Treatment	Num. Flowers	Slope (±SE)	LT_10_ * (FL at 95%)	LT_50_ * (FL at 95%)	LT_90_^ns^ (FL at 95%)	Ratio LT_50_
UTC	783	5.16 ± 0.38	−3.4 (−3.0/−3.8) a	−6.1 (−5.7/−6.4) a	−10.8 (−10.0/−11.9)	-
1000/24	830	8.72 ± 0.77	−5.4 (−5.0/−5.7) b	−7.6 (−7.3/−7.8) b	−10.6 (−10.1/−11.4)	0.80
2000/24	701	11.14 ± 0.98	−6.1 (−5.7/−6.4) c	−8.0 (−7.7/−8.2) bc	−10.4 (−9.9/−11.1)	0.76
1000/48	650	6.69 ± 0.73	−4.9 (−4.3/−5.3) b	−7.6 (−7.2/−8.0) b	−11.8 (−10.7/−13.7)	0.80
2000/48	627	7.23 ± 0.88	−5.6 (−5.0/−6.0) bc	−8.4 (−8.0/−8.9) c	−12.6 (−11.3/−15.0)	0.72
1000/96	791	5.32 ± 0.43	−3.6 (−3.2/−4.0) a	−6.4 (−6.0/−6.7) a	−11.1 (−10.2/−12.2)	0.96
2000/96	821	4.56 ± 0.37	−3.5 (−3.0/−3.9) a	−6.6 (−6.2/−7.0) a	−12.6 (−11.0/−14.5)	0.92

* LTs with the same letter are not significantly different by fiducial limits (FL) overlapping (*p* = 95%). ^ns^ No significant differences between treatments.

**Table 4:** Probit analysis results in peach and almond (experiment 2): treatments, number of fruitlets evaluated in each treatment, lethal temperatures of 10%, 50%, and 90% of flowers (LT_10_, LT_50_, and LT_90_, respectively) and ratios between LT_50_ treatments versus control.

Crop	Treatment	Num. Fruit	Slope (±SE)	LT_10_ * (FL at 95%)	LT_50_ * (FL at 95%)	LT_90_^ns^ (FL at 95%)	Ratio LT_50_
Peach	UTC	320	8.92 ± 0.34	−2.5 (−2.4/−2.7) a	−3.4 (−3.4/−3.7) a	−4.9 (−4.7/−5.2)	-
	2000/24	319	9.92 ± 0.40	−2.7 (−2.4/−2.9) a	−3.6 (−3.4/−3.8) a	−4.8 (−4.5/−5.3)	1.1
Almond	UTC	991	8.25 ± 0.39	−2.4 (−2.1/−2.7) a	−3.4 (−3.2/−3.6) a	−4.9 (−4.7/−5.3)	-
	2000/24	1035	10.02 ± 0.38	−2.9 (−2.7/−3.1) b	−3.9 (−3.8/−4.0) b	−5.2 (−5.0/−5.6)	1.1

* LTs with the same letter are not significantly different by fiducial limits (FL) overlapping. ^ns^ No significant differences between treatments.

## Data Availability

Data are contained within the article.
